# The Effect of Parasite Infection on Stable Isotope Turnover Rates of δ^15^N, δ^13^C and δ^34^S in Multiple Tissues of Eurasian Perch *Perca fluviatilis*

**DOI:** 10.1371/journal.pone.0169058

**Published:** 2017-01-03

**Authors:** Elizabeth Yohannes, Claudia Grimm, Karl-Otto Rothhaupt, Jasminca Behrmann-Godel

**Affiliations:** Limnological Institute, University of Konstanz, Mainaustrasse 252, Konstanz, Germany; University of Hyogo, JAPAN

## Abstract

Stable isotope analysis of commercially and ecologically important fish can improve understanding of life-history and trophic ecology. However, accurate interpretation of stable isotope values requires knowledge of tissue-specific isotopic turnover that will help to describe differences in the isotopic composition of tissues and diet. We performed a diet-switch experiment using captive-reared parasite-free Eurasian perch (*Perca fluviatilis*) and wild caught specimens of the same species, infected with the pike tapeworm *Triaenophorus nodulosus* living in host liver tissue. We hypothesize that metabolic processes related to infection status play a major role in isotopic turnover and examined the influence of parasite infection on isotopic turn-over rate of carbon (δ^13^C), nitrogen (δ^15^N) and sulphur (δ^34^S) in liver, blood and muscle. The δ^15^N and δ^13^C turnovers were fastest in liver tissues, followed by blood and muscle. In infected fish, liver and blood δ^15^N and δ^13^C turnover rates were similar. However, in infected fish, liver and blood δ^13^C turnover was faster than that of δ^15^N. Moreover, in infected subjects, liver δ^15^N and δ^13^C turnover rates were three to five times faster than in livers of uninfected subjects (isotopic half-life of ca.3-4 days compared to 16 and 10 days, respectively). Blood δ^34^S turnover rate were about twice faster in non-infected individuals implying that parasite infection could retard the turnover rate of δ^34^S and sulphur containing amino acids. Slower turnover rate of essential amino acid could probably decrease individual immune function. These indicate potential hidden costs of chronic and persistent infections that may have accumulated adverse effects and might eventually impair life-history fitness. For the first time, we were able to shift the isotope values of parasites encapsulated in the liver by changing the dietary source of the host. We also report variability in isotopic turnover rates between tissues, elements and between infected and parasite-free individuals. These results contribute to our understanding of data obtained from field and commercial hatcheries; and strongly improve the applicability of the stable isotope method in understanding life-history and trophic ecology of fish populations.

## Introduction

The natural abundance of stable isotopes in consumer tissues has been widely used by ecologists to identify dietary components and trophic interactions within food webs [1,2]. However the reliability of these inferences depends on knowledge on the isotopic values of the dietary items concerned and also of any isotopic turnover rates that takes place as dietary components are assimilated and incorporated into consumer tissues. In vertebrates, controlled feeding experiments have made good progress in advancing this knowledge in the last decade, (as reviewed by [3]), although, relatively few studies have attempted to examine isotopes of multiple elements in multiple tissues simultaneously.

For practical reasons, most stable isotope investigations in vertebrate consumers focus on simplified assays of a single tissue, such as muscle. Such analyses assume that muscle isotope values are a reasonable approximation of consumer isotope values overall. However, the validity of this assumption across all taxa and all individuals is increasingly questioned. Additionally, in fish, previous stable isotope analyses of parasitized tissues have indicated, somewhat controversially, that infection alters the allocation of food resources within an individual consumer ([4] and references therein).

As a rule, isotope analyses used to determine the dietary composition of a consumer presuppose that consumer tissues are maintained at isotopic equilibrium with the diet [5]. However this is not always the case, since different tissues may incorporate commonly available elements at different speed; resulting in isotopic discrepancies between tissues and between tissues and diet [6]. Estimates of consumer diets based on one-time measurements of tissue isotope values may therefore be unreliable. Laboratory controlled studies of tissue stable isotope dynamics, including measurements of tissue-specific isotope turnover rates will thus advance the accuracy of isotopic approaches in eco-physiological studies.

Parasites are known to be ecologically important stressors. Infections may alter host physiology and behavior and impact negatively on fitness and growth [7–9]. For example, the metabolic rate of vertebrate hosts can be elevated by exposure to parasites or other immune challenges [10–12]. In fish especially, parasitism is known to impact on host metabolism and this may be reflected in observable differences in stable isotope values between infected and non-infected tissues or individuals [9]. Stable isotope turnover is expected to vary with body size or age, with diet components integrated more rapidly into the tissues of fast growing juveniles than those of older specimens [3]. While parasite infection in young fish might increase metabolism, it may also direct energy and other dietary resources away from growth and into immunological defense mechanisms [13–16]. This could alter stable isotope turnover rates, though the effects, e.g. on host growth, are likely to vary greatly with host ontogenetic stage.

In this study, we performed a laboratory controlled diet-switch experiment to explore the influence of infection with the plerocercoid of the pike tapeworm *Triaenophorus nodulosus*, on tissue-specific isotope incorporation in the Eurasian perch *Perca fluviatilis* L., (hereafter perch). We examined how parasitism affects turnover of isotopes of carbon (^13^C /^12^C, shown as δ^13^C), nitrogen (^15^N /^14^N, shown as δ^15^N) and sulphur (^34^S/^32^S, shown as δ^34^S) in host liver, blood and muscle tissues. We hypothesized that the isotope signatures of more metabolically active tissues, such as blood and liver, would register the change in diet more rapidly than muscle tissue, which is thought to have a relatively slow turnover rate [17, 18]. In addition, parasite infection by increasing metabolic activity could speed up isotopic turnover even more. Turnover rates, presented in this study as isotopic half-life, are useful in studying ecological aspects of both wild and commercially cultured perch from all over Europe. If parasite infection is shown to impact tissue-specific turnover rates, value adjustments will need to be made to allow for comparison of isotopic turnover rates between infected and uninfected tissues.

Finally, we examined the general expectation that consumers will exhibit enriched stable isotope levels relative to their diets [1,6]. We calculated parasite discrimination factors (Δ_PDF_), identifying isotopic differences between host liver, blood and parasite tissues of infected animals. The parasite–host system of this study represents a specific consumer–diet pair in which the parasite depends exclusively on perch tissue as a sole dietary source and should thus represent a perfect model system to test these general expectations. Additionally it can provide first insights into turn-over rates of parasite in respect to host tissue. Prior studies on isotopic discrimination in parasite–host systems(Δ_PDF_) have however yielded controversial results whereby investigated parasites were isotopically in equilibrium, depleted or enriched relative their host (e.g., [4]). To our knowledge this is a first controlled experimental study that applied stable isotope values to stable isotope turnover rates in parasites by shifting dietary sources of the host. Indeed, parasite-host discrimination factors (Δ_PDF_), obtained from controlled laboratory studies assist to understand the mechanism for Δ_PDF_ deviations from expectations in more detail and to shade light on the physiological-interaction between host and parasite.

## Materials and Methods

### Study organisms

#### Pike tapeworm

The pike tapeworm Triaenophorus nodulosus is one of the most common cestodes in Eurasian perch and also in the American yellow perch Perca flavescens, both of which it infects as second intermediate hosts, at notable fitness cost [14,19, 20]. The detriment to host fitness may be partly attributed to the energetic costs of mounting and maintaining an immune response to infection [21, 22]. Infected adult perch in Lake Constance commonly exhibit severe hepatic disorders or other clinical manifestations [14] though these were not apparent in the young infected fish in this study. It has been shown that juvenile perch of age 42 to 152 days post hatch with a parasite intensity exceeding two parasites per liver exhibit higher mortality rates [23], while adults highly infected (more than three parasites per liver) show reduced growth [14]. The direct costs of moderate T. nodulosus infection (with a typical load of 1 or 2 parasites per liver) appear to be small, but the long-term effects and fitness consequences of such infection levels in individual fish are poorly understood.

#### Infected perch capture and husbandry

Previous studies have shown an extremely high prevalence of the pike tapeworm *T*. *nodulosus* in Lake Constance perch, with more than 90% infected within their first year of life [14, 24]. Juvenile perch (Young of the year YOY) for use as the infected study group were collected from Lake Constance (Germany) between 19^th^ and 25^th^ September 2013, using dip nets or fish traps, and stocked randomly in two separate aquaria (width = 98 cm, depth = 48 cm, height = 50 cm and volume = 235 L). Tanks were aerated and supplied with filtered (< 30 μm) lake water at a rate of 4–5 ml/s, with a 12/12 h light/dark cycle and a constant temperature of about 16.0 ± 0.1°C. In order to ensure an isotopic food resource signature consistent with that of their wild counterparts [25], infected experimental perch were supplied with zooplankton diet obtained from Lake Constance for 16 days before diet-switch experiment was conducted.

In order to minimize stress in what is known to be a highly sensitive species, handling of fish was kept to an absolute minimum. Thus no morphological measurements (length or weight) were taken before or during the diet switch experiment. Furthermore, the chemotherapy usually applied to prevent fungal infection in lake fish transferred to the laboratory was omitted to avoid any influence of treatment on the parasites.

#### Non-infected perch

The high prevalence of parasite infection in perch made it logistically difficult to obtain large numbers of non-infected fish from the wild. Given that infection could only be confirmed at the end of the experiment (i.e., after liver extraction for parasites) acquiring sufficient non-infected individuals for the comparison in the experiment would mean sacrificing an unacceptably large number of lake-caught individuals. Thus in order to generate a parasite-free control group, a cohort of perch were hatched and reared under controlled conditions in the aquarium facility of the Limnological Institute of the University of Konstanz. The rearing effort began a year in advance of the experiment.

The captive rearing protocol was as follows. Adult perch were caught in the lake at spawning time in May 2012, artificially crossed and the fertilized eggs stocked into indoor aquaria, where the larvae hatched approximately 10 days later. The larvae were raised on *Artemia* nauplii for the first week of life, then supplied with Lake Constance zooplankton sieved over 500 μm grids for the following 3 weeks. It has previously been shown that perch larvae in Lake Constance are not infected with the pike tapeworm before the age of 4–5 weeks [24]. This is due to the low level of procercoid infection in copepods during the spring season [24] and the fact that early perch larvae consume mainly nauplii and early copepodid stages too small to be infected with *T*. *nodulosus* [26]. From four weeks of age, the diet was switched to artificial pellet food (Dry food, BioMar 135 Inico 917, φ 1.5 mm) of known isotopic values for δ^15^N, δ^13^C and δ^34^S. This feeding regime permitted us to rear guaranteed *T*. *nodulosus* free perch for use as a non-infected control group in the diet switch experiment.

### Diet switch experiment and tissue sampling

All fish in both infected and non-infected (control) groups were subject to a simultaneous diet switch, starting on October 10, 2013. From the start of the experiment (day zero), all animals were placed on a diet of thawed frozen chironomids (Poseidon Aquakultur, Ruppichteroth, Germany, δ^15^N = 13.14 ± 0.12 ‰, N = 9; δ^13^C = –17.25 ± 0.06 ‰, N = 9, and δ^34^S = –2.06 ± 0.22 ‰, N = 5). These stable isotope values of the new diet differed from the pellet feed previously supplied to the captive-reared parasite-free fish by about 6 ‰, 7 ‰ and 9 ‰ for δ^15^N, δ^13^C and δ^34^S; respectively. This diet was also different from the zooplankton diet familiar to the infected lake-caught fish by approximately 2 ‰ for δ^15^N and 16.5 ‰ for δ^13^C and 9 ‰ for δ^34^S. The fish were supplied with the chironomid diet for a maximum of 50 (uninfected group) and 100 (infected group) days.

All fish were killed with an overdose of MS 222 (3-Aminobenzoic acid ethyl ester, concentration of 1 g/L water, Sigma-Aldrich, Steinheim, Germany). Fish were then promptly weighed and measured to the nearest 0.01 g and 1.0 mm, respectively, and 0.1–0.2 mL of blood were taken directly out of the heart using a pipette rinsed in heparin (0.5 mg heparin dissolved in 1 mL 0.64% NaCl, Carl Roth GmbH & Co. KG, Karlsruhe, Germany). Fish were dissected and samples of entire liver and 2 mg of filet dorsal muscle tissue were collected. From the infected group, obvious liver cysts were opened carefully and all hook-bearing *T*. *nodulosus* plerocercoids were counted and collected. Most plerocercoids were too small for individual isotope analysis, so entire infrapopulations from individual perch (between 2 and 11 worms each) were combined into a sample.

For infected fish, the experiment was conducted over 100 days, during which tissues and parasites were collected from 4 to 6 individuals on days 0, 3, 6, 9, 12, 16, 20, 27, 35, 50, 70 and 100 days after the diet switch. Because of difficulties rearing an adequate number of non-infected fish, the control group was smaller and was therefore only sampled on days 0, 3, 6, 9, 12, 16, 20, 27, 35 and 50 after the diet switch. All comparisons between infected and non-infected fish datasets were therefore restricted to the first 50 days. All fish were of the same age (one year old) and macroscopic investigation at the end of the experiment confirmed than none had reached sexual maturity. None of the non-infected fish had an infection by *T*. *nodulosus*.

### Ethics statement

This study was carried out in strict accordance with the Protection of Animals Act Germany (status 07/07/2014). The protection was approved by the regional council Freiburg (reference number 35–9185.64/1.1). All fish were killed with an overdose of MS 222 (3-Aminobenzoic acid ethyl ester, concentration of 1 g/L water, Sigma-Aldrich, Steinheim, Germany).

### Lipid extraction

Blood samples were centrifuged (at room temperature) for 20 min at 5000 rpm to separate plasma from cellular blood and to remove lipids (following [27]). Cellular blood samples were stored at –80°C until required for analysis. Dried and homogenized samples of liver (ca 2 mg) and muscle (ca. 2 mg) and dried samples of *T*. *nodulosus* (0.1 mg) were immersed in a solvent comprising 2:1 chloroform:methanol with a volume of about four times that of the sample. Samples were then mixed for 30 seconds, left undisturbed for approximately 20 min, centrifuged for 5 min at 3400 rpm, and the supernatant containing solvent and lipids was then removed. This process was repeated at least three times, until the supernatant was clear and colorless after centrifugation. After removals of the last clear supernatant, pellets were rinsed in distilled water and gently oven dried at 60°C for 48 hours.

### Stable isotope analysis

A total of 99 fish (60 infected and 39 non-infected subjects) were used for stable isotope analysis. δ^13^C and δ^15^N analyses were conducted on all parasite samples and subsamples of all three host tissues, while (due to lower sample amount and sample size) δ^34^S analysis was conducted on subsamples of blood and muscle only. Powdered sub-samples of approximately 0.7 mg– 1 mg were weighed to the nearest 0.001 mg in small tin cups, using a micro-analytical balance. Samples were then combusted in a vario Micro cube elemental analyzer (Elementar, Analysensysteme, Germany). The resulting CO_2_, N_2_ and SO_2_ were separated by gas chromatography and passed into the inlet of an Isoprime (Micromass, Manchester, UK) isotope ratio mass spectrometer (IRMS) for determination of ^13^C/^12^C, ^15^N/^14^N ratios and ^34^S/^32^S. Measurements are reported in δ-notation (δ^13^C, δ^15^N and δ^34^S) where

$$\delta =1000\;x\;\left(\frac{\text{Rsample}}{\text{Rstandard}}\right)–1\;‰,$$ (Eq.1)

relative to the Pee Dee Belemnite (PDB) standard for carbon and atmospheric N_2_ for nitrogen, in parts per thousand deviations (‰). Two sulfanilamide (Iso-prime internal standards calibrated and traceable to NBS-127 barium sulphate, δ^34^S = +20.3 ‰) and two Casein standards were placed between 8 unknowns in sequence. Internal laboratory standards indicated measurement errors (SD) of ± 0.05 ‰, 0.15 ‰ and 0.05 ‰ for δ^13^C, δ^15^N and δ^34^S, respectively.

### Stable isotope turnover and isotopic half-life

Group specific stable isotope turnover rates were calculated applying exponential fit described by Hobson and Clark [28], for three elements (δ^15^N, δ^13^C, δ^34^S) and three tissues (liver, blood and muscle). Isotopic turnover rates were estimated as an exponential function of time, thereby coupling growth and metabolic tissue turnover using the equation

δt=δeq+(δo−δeq)e−(λ)t (Eq. 2)

where

δ_t_ is the isotopic value (‰) of the fish at time t

δ_o_ is the initial isotopic value (‰) at equilibrium with the older diet

δ_eq_ is the isotopic value (‰) after equilibration with the new diet

t is the time (days)

and λ is the turnover rate (day^-1^), the mean length of time that an element is retained in a particular tissue pool (also known as isotopic half-life (days) [3]: defined as the rate at which a tissue reaches 50% equilibration with the isotopic value of a new diet, and is estimated as

t1/2 = ln(2)/*λ* Moreover, three potential turnover rate model versions were evaluated.

First (infected group), the group specific turnover rate was calculated for infected subjects over 50 days of experimental time. Second, (non-infected group) group specific turnover was calculated for non-infected subjects over 50 days, and third (both infected and non-infected group combined) turnover rates were based on pooled data of all fish used in the experiment (data obtained from non-infected fish and infected individuals). Version III provides a species specific turnover rate for perch in general, mimicking natural condition whereby both infected and non-infected individuals exist within a population feeding on isotopically varying dietary sources.

We used non-linear least squares to estimate each model’s parameter point estimates and associated standard error (SE). We calculated Akaike’s information criterion corrected for small sample sizes (AICc) scores to evaluate the relative support for each model and to determine how well exponential or linear model (δt = δeq–λ (δ0 –δeq) t) terms provided a better fit for the data.

AICc differences (ΔAICc) between the models were calculated as

ΔAICc=AICm−AIClow (Eq. 3)

where AIC_m_ is the AICc of a model and AIC_low_ is the lowest AICc of the competing model. The model with the best fit generates AICc = 0. Here parameter estimates are shown for the best fitting models only.

We compared stable isotope turnover rates to those predicted by body weight in vertebrate ectotherms, as reported in [3]. This recent review identified body weight as a strong indicator of δ^13^C, δ^15^N and δ^34^S turnover rate (r^2^ = 0.63) and applies the following equations for liver and blood or muscle

t1/2liver=0.21*ln(body weight)+2.47 (Eq. 4)

t1/2blood or muscle=0.21*ln(body weight)+3.23 (Eq. 5)

We used fish body weight at day 50 to predict turnover rates in non-infected and infected perch using these equations [3] and compared these values with turnover rates and isotopic half-life, calculated from the results of our diet switch experiment.

### Parasite discrimination factors (Δ_PDF_)

Isotopic differences between hosts and parasites (parasite discrimination factors, Δ_PDF_) during the course of the experiment were estimated by subtracting parasite isotope values from those of the respective host tissue

ΔPDF=δXP−δXH (Eq. 6)

where X is ^15^N or ^13^C for parasite (P) and host tissue (H).

We quantified relationships between number of parasites and Δ_PDF_ using a linear regression model and explored whether there is an association between Δ_PDF_ values of liver and blood.

The data were checked for normality and homogeneity of variances. All statistical analyses were performed using R (version 3.0.3) with an RStudio interface. The packages *lattice*, *lme4* and *pgirmess* were used for graphical, statistical and Kruskal-Wallis one-way analysis of variance by ranks, respectively.

## Results

### Stable isotope turnover rates and isotopic half-life

**Fig 1:**
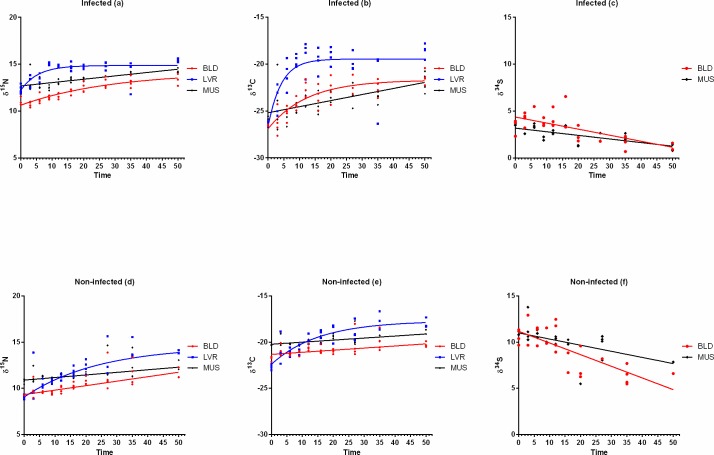
Isotopic change over time in blood (BLD), liver (LVR) and muscle (MUS) tissues in perch shown as a function of time (days) after change to isotopically distinct captive diet. Lines represent time-based exponential model fits.

Fig 1 shows δ^15^N, δ^13^C and δ^34^S turnover rates in liver, blood and muscle of European perch infected (Fig 1A–1C) with pike tapeworm *T*. *nodulosus* and non-infected subjects (Fig 1D–1F). Tables 1, 2 and 3 depict results of the comparison of Akaike’s Information criterion (ΔAICc) and parameter estimates for exponential decay functions fitted to blood, liver and muscle δ^15^N, δ^13^C, δ^34^S in infected, non-infected and pooled (infected and non-infected) samples (S1 Table). Overall, δ^15^N and δ^13^C turnover rates were fastest in liver tissue in all fish, followed by blood and muscle (Fig 1A–1F). Linear models provided the best fit for δ^34^S in blood and muscle tissues (Fig 1C and 1F). In fact, for muscle we found a linear relationship between average residence times of all three isotopes and tissue isotope values, suggesting a lack of isotopic equilibrium between muscle and diet after at least 50 days of experimental time.

**Table 1:** Comparisons of Akaike’s Information Criterion corrected for small sample size (ΔAICc) among two turnover models that treat infected and non-infected either separately (version I) or combined for each group (version II). Note models were fitted using time-based model for each isotope and tissue to either with group specific turnover rate parameter λ and δeq (Model I). E = exponential model. L = linear model. For some data sets, robust regression (coupled with outliner removal) failed to converge on a best-fit curve. For these data sets, the results shown are for ordinary least-squares regression with no outlier detection. n = sample size.

		Infected	Non-infected
	Tissue	ΔAICc -Model I (n, best-fit)	ΔAICc -Model II (n, best-fit)
δ^15^N	Blood	6.44(38, E)	-0.12(36, L)
	Liver	19.62(40, E)	22.08(36, E)
	Muscle	-2.04(40, L)	0.00(36, L)
δ^13^C	Blood	14.09(39, E)	-0.98(37, L)
	Liver	29.99(40, E)	14.58(39, E)
	Muscle	-1.90(40, L)	0(37, L)
δ^34^S	Blood	0(27, L)	-2.15(26, L)
	Muscle	-1.47(25, L)	0(22, L)

**Table 2:** Parameter estimates and standard errors (SE) the one-compartment exponential decay function fitted to blood, liver and muscle δ^15^N, δ^13^C, δ^34^S of infected (I) Eurasian Perch (*Perca fluviatilis*) over a 50 days of experiment. δ_0_ is the isotope ratio at the start of the experiment, δeq is the asymptote (plateau) of the isotope ratio, and λ is the incorporation rate of the element.

Tissue	Group	δ_0_ (SE)	δeq (SE)	λ (SE)	Half-life in days (95%CI)
**δ**^**15**^**N**					
Blood	I	10.62(0.19)	14.15(0.72)	0.03(0.01)	20.71(11.19–98.15)
Liver	I	12.20(0.33)	14.85(0.18)	0.18(0.05)	3.75(2.33–9.56)
Muscle	I	12.61(0.18)	16.59(5.45)	0.01(0.02)	59.05(12.81 - ∞)*
**δ**^**13**^**C**					
Blood	I	-26.91(0.48)	-21.70(0.45)	0.08(0.02)	8.54(5.52–19.0)
Liver	I	-26.68(0.79)	-19.46(0.37)	0.24(0.06)	2.92(1.90–6.26)*
Muscle	I	-25.45(0.46)	-25.45(3.45)	.02(0.03)	29.09(8.32-∞)*
**δ**^**34**^**S**					
Blood	I	4.36(0.43)			0.0006(0.03)
Muscle	I	3.44(0.29)	1.14(0.89)	0.04(0.03)	18.78(6.95-∞)*

* Linear regression is the overall best-fit.

**Table 3:** Parameter estimates and standard errors (SE) the one-compartment exponential decay function fitted to blood, liver and muscle δ^15^N, δ^13^C, δ^34^S of non-infected (NI) Eurasian Perch (*Perca fluviatilis*) over a 50 days of experiment. δ_0_ is the isotope ratio at the start of the experiment, δeq is the asymptote (plateau) of the isotope ratio, and λ is the incorporation rate of the element.

Tissue	Group	δ_0_ (SE)	δeq (SE)	λ (SE)	Half-life in days (95%CI)
**δ**^**15**^**N**					
Blood	NI	9.17(0.15)	13.05(1.81)	0.02(0.01)	36.44(15.11-∞)*
Liver	NI	9.05(0.18)	14.56(0.51)	0.04(0.01)	16.61(11.99–27.03)
Muscle	NI	10.89(0.12)	239.4(3517)	0.0001(0.02)	56.39(∞)*
**δ**^**13**^**C**					
Blood	NI	-21.50(0.16)	-20.30(0.37)	0.05(0.03)	14.17(5.85-∞)*
Liver	NI	-22.40(0.33)	-17.69(0.49)	0.06(0.02)	10.63(0.03–0.10)
Muscle	NI	-20.22(0.13)			0.0002(0.02)
**δ**^**34**^**S**					
Blood	NI	11.43(0.59)	0.38(13.13)	0.01(0.02)	44.11*
Muscle	NI	10.84(0.22)			0.00(0.03)

* linear regression is the overall best-fit solution.

#### Stable isotope turnover rates and isotopic half-life in infected perch

In infected perch, δ^15^N steady-state was reached only for liver tissues while δ^13^C steady-state was reached for both liver and blood tissues, Fig 1. Liver δ^15^N and δ^13^C turnover rates in infected fish were equivalent and isotopic half-life reached up to 3 days (Table 2). These calculated isotopic half-lives in the liver of infected perch are an excellent match for the 3 to 3.7 days estimated from body size alone according to the allometric scaling method [Eq 6 and 7] of [3].

In infected subjects, δ^34^S slopes of linear regression fits for blood and muscle were = –0.06 ± 0.01 and –0.04 ± 0.01, respectively, implying a relatively lower rate in sulphur-containing protein synthesis and degradation in these tissues.

#### Stable isotope turnover rates and isotopic half-life in non-infected perch

In non-infected perch, equilibrium for both δ^15^N and δ^13^C was reached in liver tissue only.

In this group, liver δ^15^N and δ^13^C half-lives were ca. 16 and 10 days, respectively, Table 3. These values are three to five times higher in infected subjects (only 3 days for both elements) compared to un-infected perch. By implication, infected perch liver δ^15^N would indicate a highly recent diet (few days) compared to uninfected perch liver which might need approximately two weeks to reach a 50% turnover in δ^15^N.

While the turnover rates for both liver δ^15^N and liver δ^13^C in infected fish were equivalent, in non-infected subjects, liver δ^13^C turnover was faster than δ^15^N (Table 3); thus, resulting in half-lives greater by about a week in δ^15^N. The isotope values of δ^34^S in blood and muscle failed to reach isotopic equilibrium with diet. In non-infected subjects, δ^34^S slopes of linear regression fits for blood showed faster turnover rate (slope = –0.13 ± 0.02) than in muscle (slope = –0.07 ± 0.02). Finally, version III turnover rates based on pooled data obtained from non-infected fish and infected individuals, representing a species specific turnover rate for perch in general is given as supplementary material (S1 Fig and S1 Table).

#### Parasite δ^15^N and δ^13^C turnover rates and discrimination factors (Δ_PDF_)

**Fig 2:**
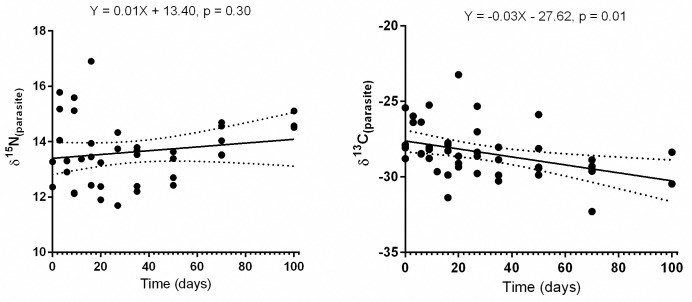
Nitrogen (δ^15^N) and carbon (δ^13^C), isotopic change over time in parasites obtained in liver tissue of perch host shown as a function of time (days) after host (fish) is shifted into to isotopically distinct diet. Lines represent time-based linear model fits (and dotted lines 95% CI).

**Fig 3:**
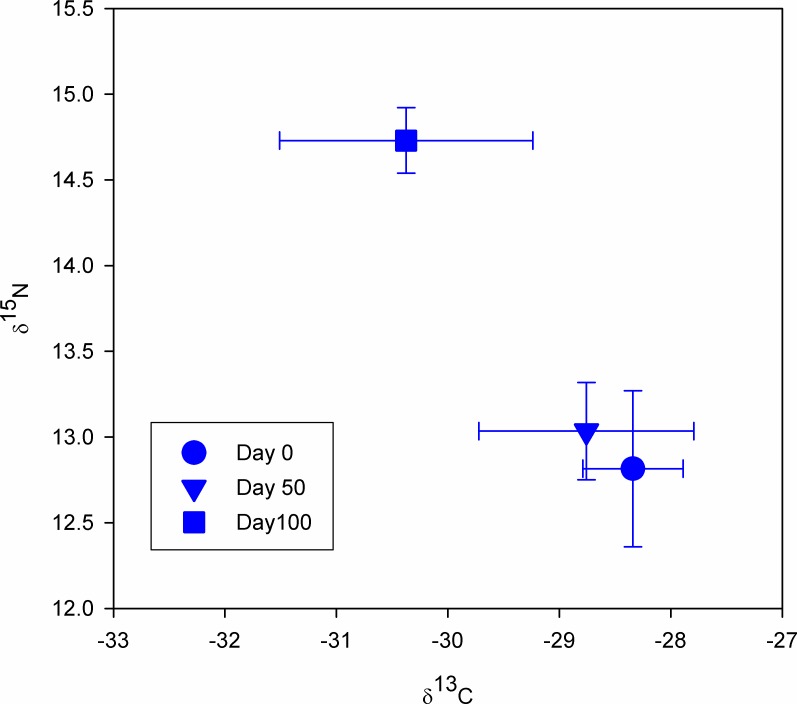
*T*. *nodulosus* Nitrogen (δ^15^N) and carbon (δ^13^C) values at day 0, day 50 and day 100, respectively.

Fig 2 shows δ^15^N and δ^13^C changes in the pike tapeworm *T*. *nodulosus* within the 100 experimental days in the laboratory. Overall, parasite δ^15^N and δ^13^C turnover rates were with linear models providing no apparent changes in parasite δ^15^N with time (slope = 0.01 ± 0.01, N = 39 per day). Yet, detailed data investigation for δ^15^N values between days 0, 50 and 100 (ANOVA followed by Bonferroni multiple comparison indicate) showed a significant step-wise enrichment in δ^15^N (Fig 3 and Table 4). For carbon isotopes, we found a significant linear change with time (slope = -0.03 ± 0.01 per day, N = 45, Fig 3) and parasite δ^13^C was more depleted by day 100 and showed a relatively higher coefficient of variation (6.48%) compared to δ^15^N (2.86%) at day 100 (Fig 3 and Table 4). This is interesting and indicates a shift of parasite tissue following dietary shift in the host and isotopic alternation in the host liver and/or blood tissue.

**Table 4:** ANOVA followed by Bonferroni multiple comparisons for *T*. *nodulosus* δ^15^N values. δ_0_, δ_50_ and δ_100_ correspond to δ^15^N value of the parasite on day 0, day 50 and day 100, respectively.

*T*. *nodulosus*			
δ^15^N	(F_2,9_ = 16.39, P = 0.02*)		
	Mean		
Date	Difference	SE	P
δ_0 -_ δ_50_	-0.22	0.45	1
δ_0 -_ δ_100_	-2.08	0.46	0.08*
δ_50 -_ δ_100_	-1.86	0.37	0.005*

*show significant values.

**Fig 4:**
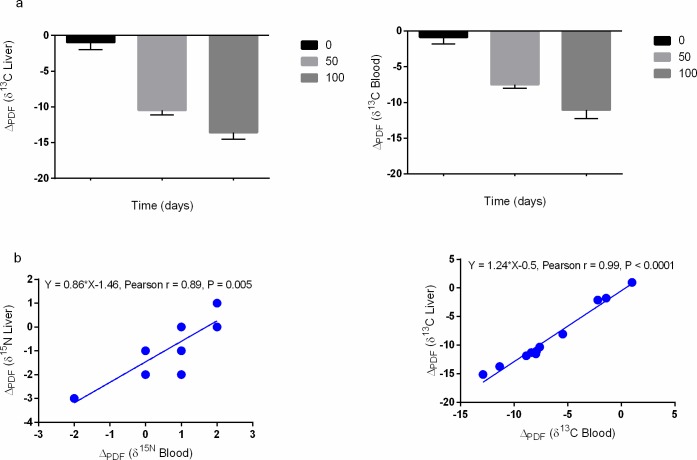
Parasite isotope discrimination factors Δ_PDF_ (a) and the correlation of elemental Δ_PDF_ in perch liver and blood (b).

We recorded most pronounced parasite-liver and parasite-blood carbon ΔPDF values that shifted from zero to negative values (Fig 4A). At the start of the experiment, liver and blood δ^13^C Δ_PDF_ values were equivalent to zero. In contrast, blood δ^15^N Δ_PDF_ values were equivalent to zero at the end of the experiment (for days 0, 50 and 100 were 2.03 ± 0.48, -0.19 ± 1.19 and 0.69 ± 0.89, respectively). This implies a shift into equilibrium with host diet during captivity in the laboratory. Finally, δ^15^N and δ^13^C Δ_PDF_ values in each tissue (liver or blood) were positively and significantly correlated (Fig 4B). S2 Table exhibits the liver and blood δ^15^N and δ^13^C Δ_PDF_ values at days 0, 50 and 100.

The prevalence of pike tapeworm infection in the lake-caught experimental perch was 88% (91% for females and 85% for males). For both females and males, the most common infection intensities were 1–2 parasites, with a maximum intensity of up to 11 tapeworms per liver recorded in two individuals (Table 5).

**Table 5:** Pike tapeworm (*Triaenophorus nodulosus*) prevalence in young of the year Eurasian Perch (*Perca fluviatilis*).

	Prevalence	Parasite	Infected fish	Parasite Intenisty
	(%)	Abundance	(n)	(Mean ±SD)
**Male**				
**Total (Infected)**	**85.19**		**23**	**3.00 ± 2.26**
	7.4	≥ 5	2	9.50 ± 2.12
	33.3	3–4	9	3.22 ± 0.44
	44.44	1–2	12	1.75 ± 0.45
**Fish examined**		**27**		
**Female**				
**Total (Infected)**	**90.91**		**30**	2.90 ± 2.28
	21.21	≥ 5	7	5.67 ± 2.19
	18.18	3–4	6	3.50 ± 0.55
	51.52	1–2	17	1.35 ± 0.49
		0	3	
**Fish examined**		**33**		

## Discussion

### Stable isotope turnover rates and half-lives

Protein metabolism in fish liver (including synthesis and degradation as well as the mechanisms that regulate these processes) is known to be one or two levels higher in order of magnitude than in muscle [29,30]. Previous findings imply that the metabolic contribution of δ^15^N and δ^13^C as elemental turnover rate in liver can reach ca. 90% [17]Overall, δ^15^N and δ^13^C turnover was faster in perch liver than in blood and muscle (Fig 1). In line with our hypothesis, and in agreement with the previous studies of [17,18], these results confirm that metabolically active tissues such as liver have higher turnover rates than metabolically less active tissues such as muscle.

Turnover rates for δ^15^N and δ^13^C in the livers of infected fish, were 3 to 4 times faster than those in non-infected livers (Table 2A and 2B), indicating a higher metabolic rate and nutrient routing in infected individuals. Fish metabolism, is subject to the influence of many factors including genetics, immunological condition and nutritional status [31]. Clearly, the impact of parasitism will depend on the severity and duration of infection. Chronic infections may affect the energy budget of the hosts, and be especially demanding in hosts whose ability to compensate for other energy demanding activities (such as growth and reproduction) is already compromised [32,33]. A positive correlation between parasite infection and metabolic rate (and higher dietary requirement) in fish has been noted in some previous studies [34] which in turn may increase turnover in infected tissues. The increased turnover rate in infected perch livers identified in the study implies a direct effect of the parasite infection on carbon and nitrogen turnover and isotopic discrimination.

Liver isotopic half-lives that we report (approximately 3–5 days) in infected subjects in this study are similar to half-lives of 3 days for δ^13^C and δ^15^ in deer mice *Peromyscus maniculatus* [35], and for δ^13^C values in chicken *Gallus gallus* [36], Japanese quail *Coturnix japonica* [28], Japanese temperate bass *Lateolabrax japonicas* [37], mouse *Mus musculus* [38]) and white-footed mouse *Peromyscus leucopus* [39].

In non-infected fish, isotopic turnover of carbon is faster than that of nitrogen (Table 3), irrespective of the tissue, indicating a greater metabolic contribution of carbon. In infected fish liver, however, turnover rates of δ^15^N and δ^13^C were equivalent. This strengthens the theory that the elemental isotopic composition of tissues (e.g. liver vs. blood) could primarily be determined by the efficiency at which these elements are incorporated into the tissue [17, 40]. Such variations in the isotopic ratios may offer a new means to study pathological conditions.

The observed variations in turnover documented here and elsewhere presumably reflect differences in the biological and chemical function of each element in different tissues [17, 37, 41, 42]. In comparison to non-infected liver, turnover rates of δ^15^N and δ^13^C in infected liver tissue were faster (half-lives of only 4 and 3 days, respectively). In contrast, half-lives of δ^15^N and δ^13^C in uninfected liver tissue were ca. 16 and 10 days, respectively. These differences implicate that the incorporation of these elements is affected by infection. This could be related to an increased demand for dietary carbon and nitrogen in order to combat parasite development or to maintain an immune response to parasitic invaders. In non-infected fish, the turnover rates of the two elements responded differently (nitrogen had a slower turnover than carbon). This indicates that the incorporation of one element is more affected by infection than the other.

Muscle turnover rate in most fishes is predominantly governed by growth and other energy routing patterns [43, 44], and the experimental duration of 100 days was insufficient time for any of the three elements investigated to reach isotopic diet-muscle equilibrium. In non-infected subjects, blood δ^34^S turnover rate was faster (ca. 0.13 day^-1^) than in infected individuals ca. 0.06 day^-1^). This confirms that blood δ^34^S turnover rate were about twice faster in non-infected individuals and implies that parasite infection might have significantly retarder δ^34^S turnover rate and the turnover of sulphur containing amino acids that may play an essential role in immune activity [45].

### Isotope turnover in pike tapeworm and implications for host fish

The stable isotope values achieved for pike tapeworm samples in this study fit neither exponential constant nor linear function terms. However careful exploration of the data strongly indicates that parasite isotope values changed substantially over the course of the100-day experiment. By the last sampling date, parasites had become enriched in heavy nitrogen but highly depleted in ^13^C compared to initial values. The depletion of the heavy ^13^C may reflect an increased incorporation of the light ^12^C resulting from an increasing lipid demand of parasite tissues during parasite growth. During lipid synthesis, the host discriminates against the heavier ^13^C isotope, thus leading to the accumulation of the lighter ^12^C isotope within fatty liver tissue [45]. When the pike tapeworm feeds on the already depleted tissue, it itself is expected to be depleted even further, as is visible in this study. These changes imply that the encysted parasites are not completely dormant and that their presence is affecting the isotopic physiology of their hosts.

We note that juvenile perch in the infected group were also likely to be infected by additional parasites, including trematodes (eye flukes) and gut cestodes, which are common in Lake Constance perch [23,24]. Although we are not aware of any visible or gross pathological expression caused by these parasite species, it cannot be ruled out that such additional infection might have contributed to the observed increase in isotopic turnover in the infected groups.

This study revealed shorter half-lives for nitrogen and carbon in tissues of infected wild perch compared to non-infected laboratory-reared fish, indicating a higher metabolic rate in infected individuals, and higher isotopic turnover in liver of infected subjects. In the natural population of perch in Lake Constance, juveniles may be infected with *T*. *nodulosus* as young as four weeks of age and by 12 weeks post-hatching, prevalence is approximately 50% [24]. This high prevalence in early life stages is indicative of chronic infection that will last for several years in most individuals. Parasitic infections may also induce malformations, impair swimming performance and affect body ion concentrations of juvenile fish [46–48]. In our study, we did not investigate for the above mentioned manifestations; however, all of these will probably decrease the individual fitness of infected fish.

Our study confirmed previous assessments of isotope turnover rates in fish blood, liver and muscle, emphasizing that liver and blood respond faster to a dietary shift than muscle tissue with liver always responding the fastest [17,18]. These significant differences in tissue-specific turnover rates clearly demonstrate the need for careful tissue choice in stable isotope studies, especially those of fishes whose life history includes regular habitat or dietary changes such as the ontogenetic shift between pelagic and littoral feeding seen in young perch. Depending on the scientific question being addressed, analyzing the isotopic signatures of different tissues may allow for the exploration of diets consumed over different time scales, for example of up to one week using liver tissue or up to 1–2 months using muscle tissue. While these results emphasize the importance of tissue selection in isotopic studies, the use of blood might be particularly interesting, as sampling can take place without sacrificing animals and allows repeated measurements.

### Parasite turnover rates and discrimination factors (Δ_PDF_)

At the start of the experiment, parasite and host isotope values in liver and blood where identical and particularly for carbon isotopes, confirming that the parasite isotope values are in equilibrium with the host tissue isotope values. Thus, it is likely that the parasites have infected the fish long time ago and the parasites reside in the liver for an extended time and these fish did not exhibit any significant shift in δ^13^C over extended period of time. By shifting the host (fish) into isotopically distinct diet, we were then able to alter the elemental isotope signatures of the liver and blood tissue. By doing so, for the first here, we were again able to shift the stable isotope values of the parasites residing in the host liver. We thus report a more depleted carbon and more enriched nitrogen isotopes in parasites on day 100 relative to day 0; and a strong positive correlation in elemental Δ_PDF_ values in each tissue. These results confirm that liver parasite *T*. *nodulosus* is able to utilize a variety of metabolic resources presumably distinct nutrients derived from both liver and blood. By implication, these parasites were not at a complete dormant physiological state. Parasites might thus play a significant role not only in affecting the tissue carbon, nitrogen or sulphur turnover rates of the liver where they reside but also other tissues such as blood and ultimately affecting the metabolic rate of the host. As such, these findings imply the hidden costs of chronic and persistent infection that may result an accumulation of seemingly minor but adverse effects that might eventually affect life-history parameters.

The most pronounced (and highly negative in value) in the parasite-liver and parasite-blood carbon isotope Δ_PDF_ values were not expected and to our knowledge have not been reported elsewhere. Since this parasitic cestodes might have a limited ability to synthesize carbohydrates de novo or to undertake gluconeogenesis (e.g., [4]), these results might imply that the parasites are obliged to retain heavier isotope containing carbohydrates required for the biosynthesis of complex products from their hosts and thus with time, resulting in lower (negative parasite-liver and parasite-blood carbon) Δ_PDF_ values. None of our elemental or tissue Δ_PDF_ results are in complement with the more positive δ^13^C and δ^15^N discrimination values as expected in conventional consumer–diet systems. Indeed, these values display interesting results for field caught fish that seasonally feed on different food sources and that the parasites-host systems may not always be understood as traditional consumer–diet systems.

In summary, we found that turnover rates in liver and blood were higher in infected fish than non-infected fish. From these observations we can infer that although infected fish have higher metabolic rates, the energy gained could be directed into anti-parasite defense mechanisms such as immune responses instead of into growth. Parameter measurements reported in this study from infected groups (as opposed to data from non-infected groups) simulate the natural condition in the field and could be applied in future field-oriented researches. Finally, we confirm that while stable isotope analysis is a powerful tool for reconstructing trophic interactions and understanding life-history patterns and feeding ecology, its use can be optimized by investigating multiple tissues (blood, liver and muscle) and multi elements (carbon, nitrogen and sulphur) approaches at a species-specific level.

## Supporting Information

S1 Fig Time-based exponential model fits for blood (BLD), liver (LVR) and muscle (MUS) tissues in perch of both infected and non-infected groups. The model provides a species specific turnover rate for perch in general, mimicking natural condition whereby both infected and non-infected individuals exist within a population feeding on sources with isotopically varying values.
(TIF)

S1 Table Comparisons of Akaike’s Information Criterion corrected for small sample size (ΔAICc) among two turnover models that treat infected and non-infected groups together. Note models were fitted using time-based model for each isotope and tissue as a single species specific turnover rate parameter λ and δeq (Version III). E = exponential model. L = linear model. For some data sets, robust regression (coupled with outliner removal) failed to converge on a best-fit curve. For these data sets, the results shown are for ordinary least-squares regression with no outlier detection. n = sample size.
(DOCX)

S2 Table δ^13^C pike tapeworm (*Triaenophorus nodulosus)—*Eurasian perch (*Perca fluviatilis*) ΔPDF values in liver and blood at days 0, 50 and 100. (DOCX)
