# Supplementary material for: The Effect of Parasite Infection on Stable Isotope Turnover Rates of δ15N, δ13C and δ34S in Multiple Tissues of Eurasian Perch Perca fluviatilis
Source: PLoS One. 2017 Jan 3;12(1):e0169058. doi: 10.1371/journal.pone.0169058 (PMC5207537; doi:10.1371/journal.pone.0169058)
Supplement: Supplementary file 2 [file pone.0169058.s003.docx]

Isot
$\delta^{15}N$
$\delta^{13}C$
